# miR-217-5p NanomiRs Inhibit Glioblastoma Growth and Enhance Effects of Ionizing Radiation via EZH2 Inhibition and Epigenetic Reprogramming

**DOI:** 10.3390/cancers17010080

**Published:** 2024-12-30

**Authors:** Jack Korleski, Sweta Sudhir, Yuan Rui, Christopher A. Caputo, Sophie Sall, Amanda L. Johnson, Harmon S. Khela, Tanmaya Madhvacharyula, Anisha Rasamsetty, Yunqing Li, Bachchu Lal, Weiqiang Zhou, Karen Smith-Connor, Stephany Y. Tzeng, Jordan J. Green, John Laterra, Hernando Lopez-Bertoni

**Affiliations:** 1 Hugo W. Moser Research Institute at Kennedy Krieger, Baltimore, MD 21205, USAssudhir1@jh.edu (S.S.); tmadhva1@jh.edu (T.M.); liyu@kennedykrieger.org (Y.L.);; 2 Department of Internal Medicine, Mayo Clinic, Rochester, MN 55905, USA; 3 Department of Biomedical Engineering, Institute for NanoBioTechnology, Baltimore, MD 21205, USAstzeng1@jhmi.edu (S.Y.T.);; 4 Translational Tissue Engineering Center, Johns Hopkins University School of Medicine, Baltimore, MD 21205, USA; 5 Department of Neurology, Johns Hopkins University School of Medicine, Baltimore, MD 21205, USA; 6 Department of Biology, Johns Hopkins University, Baltimore, MD 21218, USA; 7 Department of Biostatistics, Johns Hopkins Bloomberg School of Public Health, Baltimore, MD 21205, USA; wzhou14@jhu.edu; 8 Department of Ophthalmology, Johns Hopkins University School of Medicine, Baltimore, MD 21205, USA; 9 Department of Oncology, Johns Hopkins University School of Medicine, Baltimore, MD 21205, USA; 10 Department of Materials Science & Engineering, Johns Hopkins University, Baltimore, MD 21218, USA; 11 Department of Chemical & Biomolecular Engineering, Johns Hopkins University, Baltimore, MD 21218, USA; 12 Department of Neurosurgery, Johns Hopkins University School of Medicine, Baltimore, MD 21205, USA; 13 Sidney Kimmel Comprehensive Cancer Center at Johns Hopkins, Baltimore, MD 21231, USA; 14 Department of Neuroscience, Johns Hopkins University School of Medicine, Baltimore, MD 21205, USA

**Keywords:** GBM, miRNAs, nanoparticles, epigenetics, targeted therapies, EZH2

## Abstract

**Simple Summary:**

Cancer cells arise from multiple complementary genomic and epigenomic abnormalities that deregulate the pathways controlling cell proliferation, cell survival, and tissue homeostasis. Epigenetic modifications of histones, DNA, chromatin structure, and non-coding RNA expression are emerging as critical determinants of gene expression and essential drivers of tumor cell heterogeneity and neoplastic cell phenotypes. Multipotent stem-like cells (also referred to as cancer stem cells, CSCs) are critical determinants of tumor propagation, therapeutic resistance, and recurrence following treatment. However, our understanding of how these multidimensional epigenetic modifications cooperatively drive cancer cell stemness and how to efficaciously target them is currently inadequate to positively impact patient outcomes.

**Abstract:**

**Background/Objectives**: CSCs are critical drivers of the tumor and stem cell phenotypes of glioblastoma (GBM) cells. Chromatin modifications play a fundamental role in driving a GBM CSC phenotype. The goal of this study is to further our understanding of how stem cell-driving events control changes in chromatin architecture that contribute to the tumor-propagating phenotype of GBM. **Methods**: We utilized computational analyses to identify a subset of clinically relevant genes that were predicted to be repressed in a Polycomb repressive complex 2 (PRC2)-dependent manner in GBM upon induction of stem cell-driving events. These associations were validated in patient-derived GBM neurosphere models using state-of-the-art molecular techniques to express, silence, and measure microRNA (miRNA) and gene expression changes. Advanced Poly(β-amino ester) nanoparticle formulations (PBAEs) were used to deliver miRNAs in vivo to orthotopic human GBM tumor models. **Results**: We show that glioma stem cell (GSC) formation and tumor propagation involve the crosstalk between multiple epigenetic mechanisms, resulting in the repression of the miRNAs that regulate PRC2 function and histone H3 lysine 27 tri-methylation (H3K27me3). We also identified miR-217-5p as an EZH2 regulator repressed in GSCs and showed that miR-217-5p reconstitution using advanced nanoparticle formulations re-activates the PRC2-repressed genes, inhibits GSC formation, impairs tumor growth, and enhances the effects of ionizing radiation in an orthotopic model of GBM. **Conclusions**: These findings suggest that inhibiting PRC2 function by targeting EZH2 with miR-217-5p advanced nanoparticle formulations could have a therapeutic benefit in GBM.

## 1. Introduction

Cancer cells arise from multiple complementary genomic and epigenomic abnormalities that deregulate the pathways controlling cell proliferation, cell survival, and tissue homeostasis [1]. Epigenetic modifications of histones, DNA, chromatin structure, and non-coding RNA expression are emerging as critical determinants of gene expression and essential drivers of tumor cell heterogeneity and neoplastic cell phenotypes [2]. The cells that constitute solid malignancies vary in their capacity to propagate tumor growth and resist therapy [3]. Among these different cell subpopulations are multipotent stem-like cells (also referred to as cancer stem cells, CSCs) that are critical determinants of tumor propagation, therapeutic resistance, and recurrence following treatment [3]. Substantial evidence indicates that cancer cells are highly plastic and dynamically transition bi-directionally between stem-like/tumor-propagating cells (CSC state) and more differentiated/non-tumor-propagating states (non-CSC state) in response to contextual epigenetic events [4]. Aberrant patterns of DNA modification, histone modification, and chromatin structure ultimately regulate these phenotype transitions through deregulated gene expression [5]. However, our understanding of how these multidimensional epigenetic modifications cooperatively drive cancer cell stemness and oncogenesis is inadequate to positively impact patient outcomes.

We and others have found that the expression of a defined set of transcription factors involved in cell potency regulation is sufficient to recapitulate the neoplastic epigenetic landscape and reprogram non-tumorigenic cancer cells to display a tumor-propagating stem-like phenotype [6]. We have shown that two of these reprogramming transcription factors, Oct4 and Sox2, are sufficient to induce non-glioma-propagating non-stem-like glioma cells (non-GSCs) to transition to glioma-propagating stem cells (GSCs) [7]. The specific epigenetic pathways induced by reprogramming transcription factors that drive the acquisition and/or maintenance of GSCs remain unclear. There is considerable overlap between genes regulated by PRC2 and genes regulated by the stem cell-inducing transcription factors Oct4/Sox2 in embryonic stem cells. PRC2 is a multiprotein complex consisting of Enhancer of zeste homolog 2 (EZH2), Suppressor of Zeste 12 (SUZ12), embryonic ectoderm development (EED), and RBBP4/RbAp48, which regulates gene expression and chromatin by adding methyl groups to histone H3 on lysine 27 (H3K27me1/2/3). PRC2 supports cell stemness, multipotency, and oncogenesis by silencing target genes (e.g., differentiation pathways) through local chromatin reorganization that also impacts promoter DNA methylation. Of these subunits, EZH2 is the catalytic core while SUZ12 and EED modulate EZH2 methyltransferase activity. JARID2, another component of the PRC2 complex, co-localizes with SUZ12 on chromatin and influences PRC2 recruitment in embryonic stem cells (ESCs) [8]. EZH2, which catalyzes histone 3 lysine 27 tri-methylation (H3K27me^3^), resulting in heterochromatin formation, is a particularly important epigenetic regulator of stemness in non-neoplastic and neoplastic cells [9]. Additionally, EZH2 hyperactivity increases H3K27me^3^ and repression of gene expression and is associated with multiple cancers, including GBM [10].

MicroRNAs (miRNAs) encompass species of short noncoding RNAs, 18–25 bp in length, that also repress gene expression primarily by targeting mRNA for degradation via complementary 3′-UTR seed sequences [11]. These molecules are potent determinants of cell fate and can modify the epigenome via downstream effects on DNA methylation, histone modification, and chromatin architecture [12]. Similarly, miRNA dysregulation resulting from aberrant crosstalk between DNA and chromatin modification and fate-determining transcription factors [7,12,13,14,15] contributes to cancer, including brain cancer pathogenesis [7,13,14,15,16]. Consequently, miRNAs have emerged as promising candidates for anti-cancer therapeutics [17]. Identifying and targeting epigenetic mechanisms that function as glioma stem cell (GSC) modifiers can inform new approaches to the treatment of GBM.

The goal of this study is to identify the mechanisms activated by Oct4 and Sox2 reprogramming transcription factors that drive glioma-propagating stem cells and determine their potential as targets for developing novel anti-cancer therapeutics. We show that GSC formation and tumor propagation involve the crosstalk between multiple epigenetic mechanisms, resulting in the repression of miRNAs that regulate PRC2 function and H3K27me3. Using a combination of transcriptomic analyses, bioinformatics, and molecular approaches, we show that EZH2 is highly induced in response to GSC reprogramming factors and define a subset of putative stemness suppressor genes that are repressed in a PRC2-dependent manner. Additionally, we identify miR-217-5p as an EZH2 regulator in GBM cells and show that miR-217-5p reconstitution using advanced nanoparticle formulations re-activates the PRC2-repressed genes, inhibits GSC formation, impairs tumor growth, and enhances the effects of ionizing radiation in an orthotopic model of GBM. These findings suggest that inhibiting PRC2 function by targeting EZH2 with miR-217-5p could have a therapeutic benefit in GBM.

## 2. Materials and Methods

### 2.1. GBM Neurosphere Cell Culture

The GBM-derived neurosphere lines (GBM1A and GBM1B) were originally derived and characterized by Vescovi and colleagues [18]. The low-passage primary neurospheres were derived directly from human GBM clinical specimens and from patient-derived xenografts obtained from pathological GBM specimens obtained during clinically indicated surgeries at Johns Hopkins Hospital using established methods. The induced GSC cell lines, A172-Oct4/Sox2, were developed and characterized previously by us [7]. All the neurospheres were cultured in serum-free conditions using Stemline(R) Neural Stem Cell Expansion Medium (Sigma-Millipore, Burlington, MA, USA) supplemented with 20 ng/mL epidermal growth factor (EGF) and 10 ng/mL fibroblast growth factor (FGF). The human embryonic kidney 293FT (HEK293FT) cell line and GBM cell line A172 were obtained from the ATCC and maintained in Dulbecco’s modified Eagle/F12 medium (1:1, *v*/*v*) supplemented with 10% FBS (Fetal Bovine Serum, Thermo Fisher Scientific Inc.—Waltham, MA, USA). All the cells were grown at 37 °C in a humidified incubator with 5% CO_2_. All the cell lines used in the study were tested for mycoplasma and were STR profiled.

For the extreme limiting dilution assays (ELDAs) [19], tumor cells were cultured in neurosphere medium containing EGF/FGF at decreasing cell densities ranging from 100 to 12.5 cells, with over 24 technical replicates for each dilution. For the experiments involving the indicated transgenic miRNA expression, the neurospheres were first transduced with lentivirus expressing the indicated miRNA hairpin prior to dilution. The number of wells containing spheres was counted after 14 days, and the online ELDA tool (http://bioinf.wehi.edu.au/software/elda/, last accessed on 18 December 2024) was used to calculate stem cell frequencies.

### 2.2. qRT-PCR and miRNA Expression

Total RNA was extracted from the cells using the RNeasy mini kit (Qiagen—Germantown, MS, USA). cDNA was made by reverse-transcribing 1 μg of total RNA using MuLV Reverse Transcriptase and Oligo (dT) primers (Applied Biosystems—Waltham, MA, USA). qRT-PCR was performed with a Bio-Rad CFX detection System (Bio-Rad—Hercules, CA, USA), and the expression of target genes was measured using the Power SYBR green PCR kit (Applied Biosystems). The samples were amplified in triplicate, and relative gene expression was analyzed using Bio-Rad CFX manager software v3.1 and normalized to 18S RNA. The primer sequences used to measure the expression of reprogramming transcription factors, stem cells, and neural lineage markers were previously reported by us [7]. The primer sequences used in this study were obtained from PrimerBank (https://pga.mgh.harvard.edu/primerbank/, last accessed on 18 December 2024) and are listed in Table S1.

To measure the levels of precursor miRNAs (pre-miRNAs), total RNA was extracted using miRNeasy (Qiagen), and cDNA was synthesized from total RNA (1 μg) using gene-specific primers and the High-Capacity cDNA reverse transcription kit (Applied Biosystems). The gene-specific primers included a mixture of 10 µM of each of the antisense primers with the selected miRNAs (e.g., miR-217-5p) and U6 RNA. Following an 80 °C denaturation step and 60 °C annealing, the cDNA was reverse transcribed for 45 min at 60 °C. cDNA was diluted at 1:20 prior to detection using the Power SYBR green PCR kit from Applied Biosystems. The primer sequences can be found in Table S1.

### 2.3. Chromatin Immunoprecipitation

Chromatin immunoprecipitation assays were performed using the MAGnify Chromatin Immunoprecipitation system (Life Technologies—Grand Island, NY, USA). Immunoprecipitation was performed with anti-H3K27me3 (Active Motif—Carlsbad, CA, USA) or anti-IgG (Life Technologies). Specific regions were quantified by qRT-PCR using the primers described in Table S1.

### 2.4. Lentivirus Generation and Cell Transduction

To produce lentiviral particles, we used the 2nd-generation lentiviral system according to the Addgene instructions, using the psPAX2 packaging plasmid and pMD2.G envelope plasmid (Addgene—Cambridge, MA, USA). Co-transfection of the lentiviral packaging/envelope plasmids and transfer vector into the HEK239FT (2 × 10^7^ cells/transfection) was performed using the Lipofectamine 3000 (ThermoFisher Scientific), scaled according to the manufacturer’s recommendations. After overnight incubation, sodium butyrate (Cayman Chemical—Ann Arbor, MI, USA) was added at a final concentration of 10 mM to increase the viral titer. The lentiviral particles in the supernatant were collected at 48–72 h and used to transduce cells. GBM neurospheres (1.5 × 10^5^ cells) were seeded in a 6-well cell culture plate and infected overnight with lentiviral medium containing viral particles and polybrene (1 µg/mL), supplemented with the appropriate medium. On the following morning, the cells were pelleted by centrifugation and resuspended in fresh neurosphere medium. Plasmids for Oct4 (EX-Z0092-Lv156), Sox2 (EX-T2547-Lv160), miR-217-5p mimic (HmiR0031-MR03-B), and miR217 sponge (HmiR-AN0325-AM03-B) were purchased from Gencopoeia (Rockville, MD, USA).

### 2.5. Immunoblotting

Western blot was performed using a quantitative Western blot system (LI-COR Bioscience—Lincoln, NE, USA) following the manufacturer’s instructions. The cells were lysed in RIPA buffer (Sigma-Millipore—Burlington, MA, USA) for 30 min on ice. Samples containing identical amounts of protein (25–40 µg) were resolved by NOVEX 4–12% Tris-glycine gradient gel (Thermo Scientific), transferred to Amersham Protran nitrocellulose membrane (GE HealthCare—Chicago, IL, USA), and blocked in Li-COR blocking buffer. Membranes were probed with the antibodies listed in Table S2. Secondary antibodies were labeled with IRDye infrared dyes (LI-COR Biosciences), and protein levels were quantified using the Odyssey Infrared Imager (LI-COR Biosciences). Densitometry analysis was performed using the Image Studio™ v 6.0 acquisition software from LI-COR imaging systems. Protein expression was normalized to the loading control (i.e., GAPDH or H3). A list of the antibodies used in this study can be found in Table S2.

### 2.6. Bisulfite Sequencing

Genomic DNA was isolated using the QIAamp DNA mini kit (Qiagen), and the DNA was subjected to bisulfite treatment using the EZ DNA methylation kit (ZYMO Research—Irvine, CA, USA). The bisulfite-converted DNA was amplified using the primers described by Xi et al. [20]. The PCR products were cloned into pCR II TA vectors using the TOPO-TA cloning kit (Invitrogen, Waltham, MA, USA) and sequenced using Sanger sequencing. The sequencing data were analyzed using BISMA software (http://biochem.jacobs-university.de/BDPC/BISMA/, last accessed on 24 May 2019).

### 2.7. Intra-Cranial NanomiR Delivery and Tumor Formation In Vivo

A transcranial cannula was placed so that the tip was in the right caudate/putamen of female athymic nude NCR Nu/Nu mice (8 weeks old). One week after cannula placement, the animals received 1.0 × 10^4^ GBM1A tumor-propagating cells via the cannula and were assigned into different treatment groups in a non-blinded, randomized manner. Using the same cannula, the control cohort received nanomiRs loaded with control miRNA labeled with Dy547, and the experimental group received nanomiRs loaded with the miR-217-5p mimic. The stainless steel guide and dummy cannulas were custom-ordered from PlasticsOne (Roanoke, VA, USA). The guide cannula (26-gauge) was designed to have a Decon^®^ mesh under the pedestal, and 3 mm was cut from the mesh. The guide cannula was capped with a 6.5 mm long screw-on dummy cannula so that a 0.5 mm projection extended past the guide to prevent blockage. Prior to surgical placement of the cannulas, the mice were anesthetized using a Ketamine (100 mg/kg)/Xylazine (10 mg/kg) cocktail and mounted on a stereotactic frame. A rostro-caudal incision was made with a scalpel, the skin was spread apart, the surface of the skull was exposed, and cannulas were placed at the following coordinates: AP (antero-posterior) 0.0 (0 mm from bregma), L (lateral) 1.8 (1.8 mm right from mid-sagittal line).

Control Cy3 miRNA (CP-004500-01-05) or miR-217-5p (CP-004500-01-05) was purchased from Horizon Discovery Ltd. (Cambridge, UK), and nanomiRs were prepared as previously described by us [21]. Briefly, the nanomiRs were formed in 25 mM NaAc buffer, pH 5.0, by mixing solutions of R646 polymer and miRNA in a 1:1 ratio. NanomiRs for all the lyophilized samples were formulated using the same polymer to miRNA *w*/*w* ratio as in all earlier studies (150 *w*/*w*), and the total polymer concentration was 5 mg/mL to enable a higher dose to be delivered in the limited cannula injection volume. Endotoxin-free sucrose, initially dissolved at 500 mg/mL, was then added to the nanomiRs for a final concentration of 30 mg/mL sucrose as a cryoprotectant. The nanomiRs were then aliquoted to tubes and frozen at −80 °C and lyophilized overnight at ~20 Pa and −45 °C.

For in vivo utilization, lyophilized nanomiRs were resuspended using deionized water to a final polymer concentration of 16.7 mg/mL and a final isotonic sucrose concentration of 100 mg/mL. The resuspended nanomiRs were slowly infused (5 μL) into the brains (0.5 μL/min with a 2 min wait at the end) twice a week as described for each experiment. At the end of the experiment, the animals were anesthetized and then euthanized by perfusion using 4% paraformaldehyde (PFA) according to methods approved by the Animal Use and Care Committee at Johns Hopkins University. All the sectioning and histological analysis was performed in-house. Whole brains were collected and soaked in 4% PFA for 2 days then washed 1× with PBS and soaked in 30% sucrose overnight at 4 °C then flash-frozen using dry ice. The brains were embedded in Tissue-Tek^®^ O.C.T. Compound (VWR, Radnor, PA, USA), and 20 μm sections were cut using the CryoStat system from Microm (Walldorf, Germany). All the tumor sections were analyzed by a neuropathologist in a blinded fashion.

For the ionizing radiation experiments, a subset of animals received radiation either alone or in combination with the nanomiR therapy. Radiation was administered starting 8 weeks after tumor cell implantation. The tumor-bearing mice were gently restrained in a 50 mL ventilated plastic centrifuge tube encapsulated in lead cylinders to protect normal body parts from radiation. This ensures that only the tumor-bearing brain is irradiated. The animals received 300 cGy (or sham irradiation) once a week for 2 weeks using a collimator 137Cs source. These radiation doses were without adverse side effects.

Tumor growth inhibition was determined by computer-assisted morphometric quantification of the tumor area in H&E-stained histologic sections using ImageJ software v 1.53a and volumes calculated using volume = (square root of maximum cross-sectional area)^3^. The data for all the in vivo experiments are shown as the mean tumor area distribution of all the animals used in the study.

### 2.8. RNA-Seq Analysis

RNA-Seq libraries were constructed from messenger RNA (mRNA) purified from total RNA using poly-T oligo-attached magnetic beads. After fragmentation, the first strand cDNA was synthesized using random hexamer primers, followed by the second strand cDNA synthesis using dUTP. The library was checked with the Qubit 4 Fluorometer (Thermo Fisher Scientific), real-time PCR for quantification, and a bioanalyzer for size distribution detection. The quantified libraries were pooled and sequenced on Illumina platforms followed by clustering of the index-coded samples according to the manufacturer’s instructions. After cluster generation, the library preparations were sequenced on an Illumina platform and paired-end reads were generated. An index of the reference genome (i.e., hg38) was built, and the reads were aligned to the reference genome using Hisat2 v2.0.5. Differential expression analysis of two conditions/groups (two biological replicates per condition) was performed using the DESeq2 R package (1.20.0).

### 2.9. Patient Databases

Clinical and transcriptomic data from control and glioma patient samples for EZH2 were retrieved from the GlioVis database (http://gliovis.bioinfo.cnio.es/, last accessed on 18 December 2024). EZH2 expression and survival correlations were determined in primary, IDH wild-type GBM patients. For statistical analysis, pairwise comparisons between group levels with corrections for multiple testing (*p*-values with Bonferroni post hoc test) were used. Statistical significance was calculated using an unpaired, non-parametric, student *t*-test with a Mann–Whitney post hoc test.

### 2.10. Statistical Analysis

All the experiments were performed in triplicate and repeated at least twice in each cell model (*n* ≥ 6). PRISM GraphPad 10 was used to perform all the statistical analyses presented. Two group comparisons were analyzed for variation and significance using a two-tailed, type 1 *t*-test and *p* values lower than 0.05 were considered significant and were symbolized by an asterisk in the graphs. One-way or two-way ANOVA and Tukey or Bonferroni post hoc tests were used to analyze the relationships when comparing multiple variables, with *p* values lower than 0.05 considered to be statistically significant. All the data shown are representative of means ± S.D. of triplicate results unless otherwise specified.

## 3. Results

### 3.1. GSC Induction Associates with PRC2 Activation and Repression of PRC2-Target Genes

**Figure 1:**
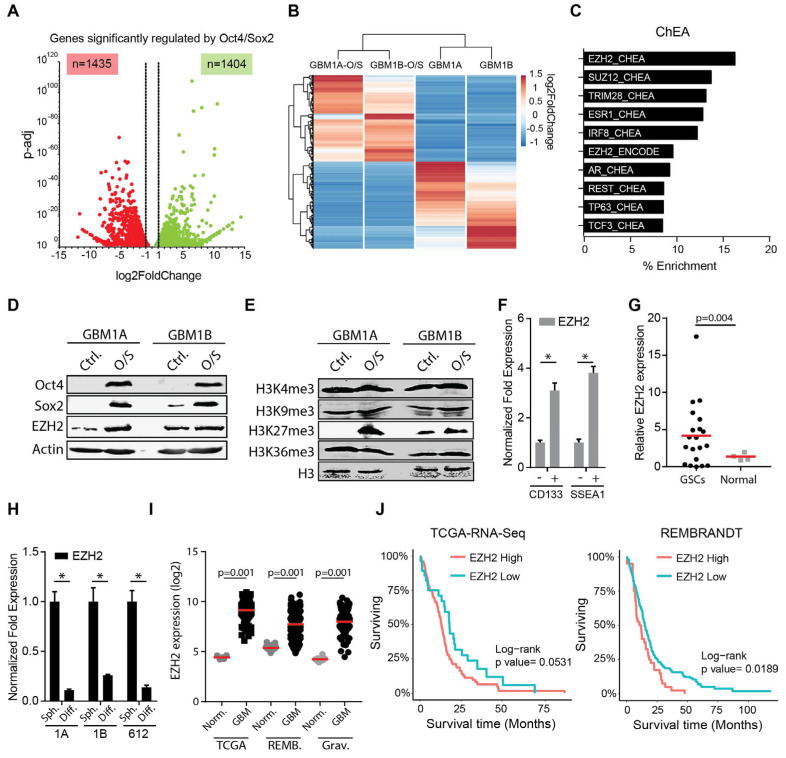
Transcriptome analysis identifies the PRC2 complex as a downstream target of Oct4 and Sox2 in GSCs. (**A**) Volcano plot comparing genes that are upregulated in Oct4/Sox2-overexpressing cells. Number of genes correspond to those that have a fold change larger than 1 or −1 with an adjusted *p* value less than 0.05. (**B**) Heatmap highlighting significant genes that are up- or downregulated after overexpression of Oct4 and Sox2 in two cells lines. (**C**) Gene set enrichment analysis showing genes downregulated in the setting of transgenic Oct4 and Sox2 expression. (**D**) Western blot analysis showing expression of Oct4, Sox2, and EZH2 protein in GSCs expressing transgenic Oct4 and Sox2. Actin used as a loading control. (**E**) Western blot showing histone modifications in GSCs expressing transgenic Oct4 and Sox2. H3 used as loading control. (**F**) qRT-PCR measuring EZH2 expression in GSCs after sorting for cells based on expression of the stem cell markers CD133 and SSEA1. (**G**) qRT-PCR analysis to measure expression of EZH2 in primary GSC isolates (*n* = 20) and glial progenitor cells (*n* = 4). (**H**) EZH2 expression in three primary GBM cells lines (GBM1A, GBM1B, and 612) after growth in neurosphere media (Sph.) or serum containing media (Diff). (**I**) EZH2 expression in normal CNS tissue vs. GBM tissue in the TCGA, Rembrandt (REMB) or Gravendeel (Grav.) datasets (**J**) Kaplan–Meier survival curves for patients with tumors expressing high or low levels of EZH2 in the TCGA, Rembrandt or Gravendeel databases. Two sample *t*-tests used to test for differences in (**F**–**H**). Log-rank test used to test for differences in (**J**). * denotes *p*-value < 0.05. Original Western blot figures can be found in Supplementary File S1.

Reprogramming transcription factors (e.g., Oct4 and Sox2) are over-expressed in neoplastic stem-like cells, including GSCs, and function to generate and/or maintain the cancer stem-like phenotype [6,22]. The stem-like phenotype coordinated by these reprogramming transcription factors drives dynamic cellular transitions within tumors by multiple coordinated events involving changes in DNA methylation, histone marks, chromatin states, and miRNA networks [7,15,21]. To further understand the downstream transcriptional effects of these reprogramming events in GBM cells, we performed RNA sequencing (RNA-Seq) in GBM neurospheres with and without the expression of transgenic Oct4 and Sox2, two potent drivers of the stem cell phenotype in GBM [7]. This analysis uncovered 1404 upregulated and 1435 downregulated genes that changed significantly in two distinct GBM neurosphere models (*p*-adj < 0.05, log fold change > 1 or <−1) (Figure 1A,B). Gene set enrichment analysis (GSEA) revealed alterations in the genes overlapping with CNS tissue and CNS cancer cell lines, as well as the signaling pathways, such as interferon signaling, TNF signaling, and the metabolic pathways, which are deregulated in multiple malignancies [23,24,25,26] (Figure S1). The promoters of genes bound by EZH2 and SUZ12, which are associated with regulation in human embryonic stem cells, were the top target genes repressed in the context of Oct4/Sox2-induced stemness in GSCs, implicating the PRC2 complex in inducing and maintaining the GSC phenotype (Figure 1C). Consistent with these bioinformatic predictions, we found that Oct4 and Sox2 co-expression induced the EZH2 protein and H3k27me3 in patient-derived GBM neurospheres (Figure 1D,E). We also detected elevated EZH2 expression in CD133^+^ and SSEA^+^ GSC subsets relative to the CD133^−^ and SSEA^−^ cell counterparts isolated from GBM1A parental cells (Figure 1F) and in primary GSC isolates compared to non-neoplastic neural stem cells (Figure 1G). Conversely, forced differentiation of three distinct patient-derived GSC isolates robustly decreased EZH2 expression (Figure 1H). To explore the relevance of these in vitro findings associated with elevated EZH2 in Oct4/Sox2-expressing GSCs, we queried three distinct clinical databases and found consistently increased EZH2 expression in GBM tissue compared to non-neoplastic controls (Figure 1I). High EZH2 expression was also associated with decreased patient survival (Figure 1J).

**Figure 2:**
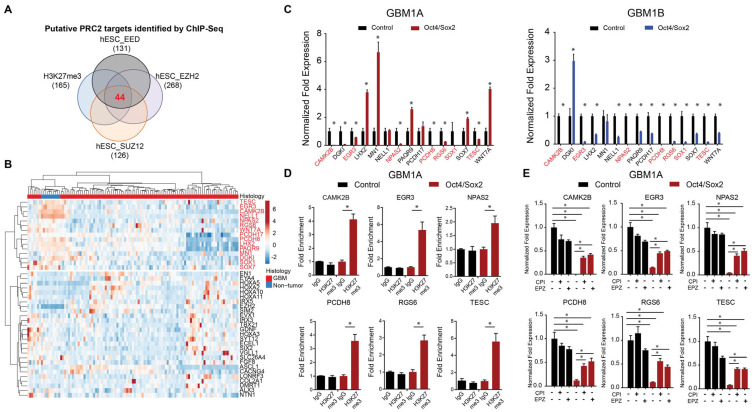
Oct4/Sox2 represses a subset of genes associated with tumor suppression in a PRC2-dependent manner. (**A**) Venn diagram showing intersection of genes directly bound by EZH2, EED, SUZ12, and H3K27me3 in human embryonic stem cells. (**B**) Heatmap of RNA-seq expression from TCGA (HG-U133A) showing expression of the 44 genes identified in (**A**). Fifteen genes highlighted in red are downregulated in GBM compared to non-tumor tissue. (**C**) qRT-PCR analysis showing expression of the 15 predicted PRC2 targets in GSCs expressing transgenic Oct4 and Sox2. Genes highlighted in red show consistent changes in 2 distinct GSC isolates. (**D**) ChIP-PCR for H3K27me^3^ at the promotor region for six punitive PRC2 targets in GSCs expressing transgenic Oct4 and Sox2. GSCs expressing GFP were used as controls. (**E**) qRT-PCR for predicted PRC2 targets with and without the EZH2 inhibitors CPI and EPZ in control vs. Oct4/Sox2 overexpression in the primary GBM cell line GBM1A. Two sample *t*-tests used to test for differences in (**C**,**D**). ANOVA with a post hoc Tukey’s test used to test for significance in (**E**). * denotes *p*-value < 0.05.

To identify candidate the PRC2 gene targets regulated by Oct4/Sox2 expression, we performed a set distribution analysis of genes that were both repressed by Oct4 and Sox2 expression in GBM neurospheres and previously identified to be direct PRC2 targets in human embryonic stem cells (Figure 1C and Figure 2A). This analysis identified 44 high-confidence PRC2 target genes, 15 of which were found to also be downregulated in the TCGA GBM transcriptomic datasets compared to non-tumor tissue (Figure 2B). qRT-PCR analysis confirmed that 7 of these 15 genes (CAMK2B, EGR3, NPAS2, PCDH8, RGS6, SOX1, and TESC) were consistently downregulated by Oct4/Sox2 across two distinct patient-derived GBM neurosphere models (Figure 2C).

Additionally, PRC2 histone mark H3K27me3 was found to be enriched in response to Oct4/Sox2 expression within 2Kb of the transcription start sites in six of the seven Oct4/Sox2 repressed genes (Figure 2D and Figure S2B). To determine whether these six putative “GSC suppressor genes” were regulated by EZH2, we treated GBM neurosphere cells ± transgenic Oct4 and Sox2 with selective pharmacological EZH2 inhibitors (CPI-1205 and EPZ-6438). EZH2 inhibition rescued the expression of all six genes (CAMK2B, EGR3, NPAS2, PCDH8, RGS6, and TESC) from repression by Oct4 and Sox2 (Figure 2E and Figure S2A). Notably, tumor-suppressive roles have been reported for CAMK2B, EGR3, NPAS2, PCDH8, and RGS6 [27,28,29,30]. These results show that EZH2 and PRC2 activity (i.e., H3K27me3) is enriched in GSCs, induced by GSC reprogramming transcription factors and epigenetically regulated GSC gene expression.

### 3.2. miR-217-5p Expression Is Regulated by DNA Methylation and Controls EZH2 Expression in GSCs

**Figure 3:**
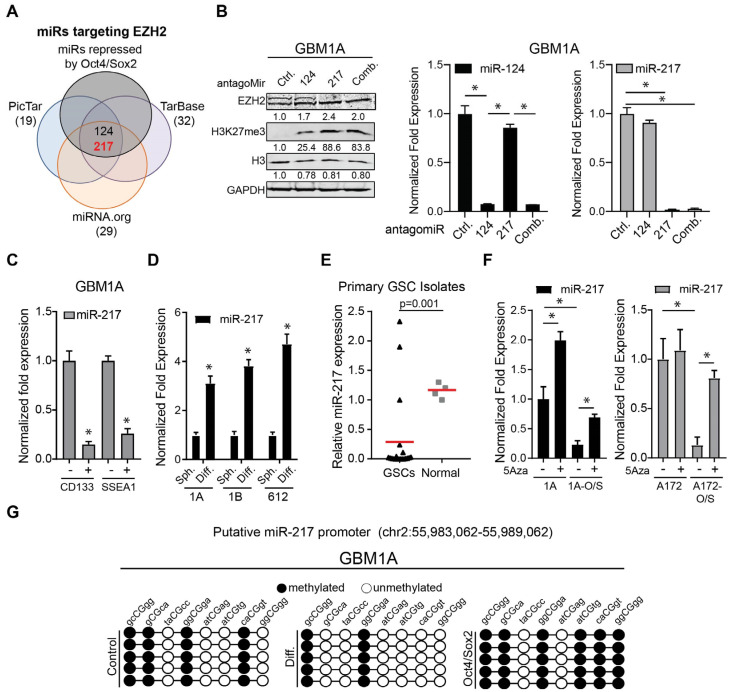
miR-217-5p expression is regulated by DNA methylation in GBM neurospheres. (**A**) Venn diagram showing miRNAs predicted to target EZH2 from PicTAR, miRNA.org, and TarBase that overlap with miRNAs repressed by Oct4 and Sox2. (**B**) Western blots measuring EZH2, H3, and H3K27me^3^ after treatment with antagomiRs to miR-124, miR-217-5p, or in combination, respectively. GAPDH and H3 were used as loading controls (left panel). qRT-PCR to measure expression of miR-124 or miR-217-5p after treatment with antagomiRs (right panel). (**C**) Expression of miR-217-5p in cells that are positive for the stem cell markers CD133 or SSEA1. (**D**) Expression of miR-217-5p in GSCs after growth in neurosphere media (Sph.) or serum-containing media (Diff). (**E**) miR-217-5p expression in primary GSC isolates (*n* = 20) and glial progenitor cells (*n* = 4). (**F**) Expression of miR-217-5p after treatment with the DNMT inhibitor 5-azacytidine (5-aza) in neurospheres expressing transgenic Oct4 and Sox2. (**G**) Methylation pattern of the putative miR-217-5p promoter via bisulfate sequencing in cell grown in neurosphere media (left panel), serum-containing media (Diff.) (middle panel), or expressing transgenic Oct4 and Sox2 (right panel). ANOVA with a post hoc Tukey’s test used to test for significance in (**B**,**F**). Two sample *t*-tests used to test for differences in (**C**–**E**). * denotes *p*-value < 0.05. Original Western blot figures can be found in Supplementary File S1.

We have previously shown that Oct4/Sox2-driven reprogramming events that induce the GSC phenotype involve induction of DNMTs, leading to repression of multiple miRNAs [7]. We hypothesized that the molecular events that drive GBM cell stemness regulate EZH2 expression and PRC2 function, in part, through a similar mechanism. We analyzed the miRNAs previously identified by us to be regulated by Oct4/Sox2 in GBM neurospheres [7] using three miRNA target prediction platforms and identified miR-124-3p and miR-217-5p as high-confidence regulators of EZH2 (Figure 3A). Consistent with this prediction, inhibiting either miR-124-3p or miR-217-5p in GBM neurospheres increased EZH2 protein levels concurrently with the corresponding increases in H3K27me3 (Figure 3B). The subsequent experiments focused on miR-217-5p since (i) both EZH2 and H3K27me3 were more robustly induced following miR-217-5p inhibition compared to miR-124-3p inhibition, and (ii) a role for miR-124-3p (i.e., a tumor suppressor function) has been previously described in GBM [31]. We examined the relationship between miR-217-5p expression and the GBM stem cell phenotype by measuring miR-217-5p levels in CD133^+^ and SSEA1^+^ GSC subsets. We found substantially lower levels of pre-miR-217-5p in the CD133+ and SSEA1+ cell subsets than in their CD133^-^ and SSEA1^-^ counterparts isolated from GBM1A parental cells (Figure 3C). This is consistent with the elevated levels of EZH2 expression in the CD133^+^ and SSEA^+^ cells described above (Figure 1F). Conversely, forced differentiation of three distinct patient-derived GSC isolates induced a 3-5-fold increase in miR-217-5p expression (Figure 3D); these conditions are associated with a ~90% decrease in EZH2 expression (Figure 1G). Lower levels of pre-miR-217-5p were also found in the primary GSC isolates compared to the non-neoplastic controls (Figure 3E).

The pan-DNA methyl-transferase inhibitor 5-azacytidine (5-Aza) induced pre-miR-217-5p in neurosphere cells expressing transgenic Oct4 and Sox2 and also induced pre-miR-217-5p levels in one of the two parental controls, implicating transcriptional repression in response to promoter DNA methylation (Figure 3F). This was confirmed using bisulfite sequencing that revealed an increase in miR-217-5p promoter CpG methylation from 50% to 75% in neurosphere cells expressing transgenic Oct4 and Sox2 compared to parental control cells and a decrease in CpG methylation from 50% to 25% upon neurosphere differentiation (Figure 3G). These results show that miR-217-5p expression is downregulated via DNA methylation and implicate EZH2 as a miR-217-5p target in GSCs.

**Figure 4:**
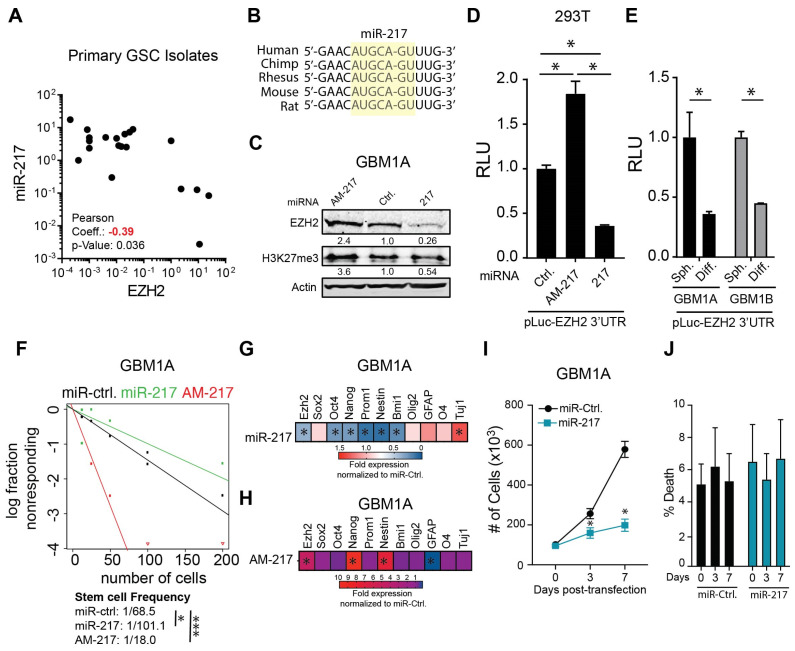
miR-217-5p regulates EZH2 expression and the stem cell phenotype in GSCs. (**A**) Correlation analysis of miR-217-5p and EZH2 expression in primary GSCs isolates. (**B**) Schematic showing conserved miR-217-5p binding sites in the 3′UTR of EZH2. (**C**) Western blots to measure EZH2 and H3K27me^3^ protein levels 3 days after GSCs were transfected with an antagomiR against miR-217-5p or miR-217-5p mimic. Actin used as a loading control. (**D**) Luciferase assay 48 h after co-transfecting a reporter containing the 3′-UTR of EZH2 with an antagomiR against miR-217-5p (AM-217) or miR-217-5p mimics (217). (**E**) Expression of luciferase-containing reporter assay with the 3′-UTR of EZH2 in two primary GSC isolates 3 days after forced differentiation. (**F**) Limiting dilution assay to measure stem cell frequency in GSCs after treatment with an antagomiRs against miR-217-5p (AM-217) or miR-217-5p mimics (217). (**G**) qRT-PCR expression of EZH2, stem cell, and neuronal lineage markers 5 days after treatment with miR-217-5p mimic (**G**) or amiR-217-5p antagomir (**H**). Equal numbers of cells were dissociated into single-cell suspensions and cultured in neurosphere medium after transfection with either miR-217-5p mimic or a control miRNA. Cells were counted at the indicated intervals using Trypan blue exclusion to measure total number of cells (**I**) and viability (**J**). ANOVA with a post hoc Tukey’s test used to test for significance in (**D**). Two sample *t*-tests used to test for differences in (**E**,**G**,**H**). Two-way ANOVA with post hoc Bonferroni test used to test for significance in (**I**). * denotes *p*-value < 0.05; *** denotes *p*-value < 0.001. Original Western blot figures can be found in Supplementary File S1.

We found a significant inverse relationship between miR-217-5p and EZH2 expression levels in primary neurosphere isolates that was consistent with EZH2 targeting by miR-217-5p (Figure 4A). The sequence analysis of the 3′UTR of EZH2 mRNA identified miR-217-5p binding sites conserved across species, predicting direct targeting (Figure 4B). EZH2 protein expression and H3K27me3 were both increased in response to miR-217-5p inhibition using an antagomir (AM-217). Conversely, both EZH2 and H3K27me3 were decreased following transfection with a miR-217-5p mimic (Figure 4C). To rigorously characterize the direct functional interactions between miR-217-5p and the EZH2 transcript, we transfected a luciferase reporter cloned with the 3′UTR region of EZH2 containing the predicted miR-217-5p binding site into 293T cells. Co-expressing miR-217-5p significantly reduced luciferase expression, and blocking miR-217-5p with antagomir enhanced luciferase expression (Figure 4D). To test whether miR-217-5p regulates EZH2 expression under physiological conditions, the GBM neurospheres were transfected with the luciferase reporter constructs and forced to differentiate. The luciferase levels were substantially inhibited in response to differentiation (Figure 4E), a condition that induces miR-217-5p expression (Figure 3D). Moreover, inhibiting miR-217-5p increased the GBM neurosphere self-renewal capacity and GSC frequency while transgenic expression of a miR-217-5p mimic decreased self-renewal and GSC frequency (Figure 4F and Figure S3A). As predicted by these results, the miR-217-5p mimic inhibited the expression of stem cell markers and EZH2 (Figure 4G,H and Figure S3C) and inhibiting miR-217-5p increased the expression of stem cell markers and EZH2 (Figure 4G,H and Figure S3B). Additionally, miR-217-5p expression significantly impaired the proliferation capacity of GBM neurosphere cells without affecting cell viability (Figure 4I,J). These data support a mechanism by which miR-217-5p regulates the GSC phenotype through its capacity to directly modulate EZH2 expression, H3K27 trimethylation, and downstream epigenetic gene expression.

### 3.3. In Vivo Delivery of miR-217-5p Mimics Inhibits Growth of Orthotopic GBM Xenografts and Sensitizes Them to Ionizing Radiation (IR)

**Figure 5:**
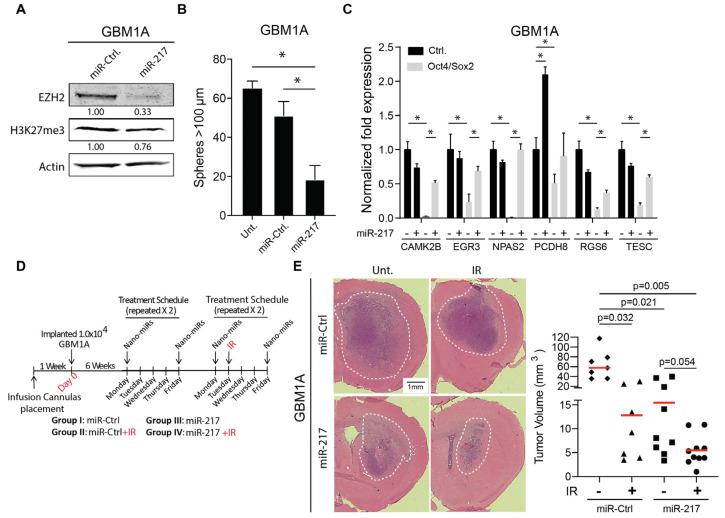
miR-217-5p nanomiRs inhibit GBM growth and enhance effects of IR. (**A**) Western blot analysis showing EZH2 and H3K27me3 expression 3 days after miR-217-5p nanomiR transfection. (**B**) Sphere formation assay 14 days after GSCs received control or miR-217-5p nanomiRs. Untreated GSCs were included as a control. (**C**) qRT-PCR for predicted tumor suppressor PRC2 targets 4 days after GSCs received control or miR-217-5p nanomiRs. (**D**) Schematic of in vivo nanomiR treatment +/− ionizing radiation (IR). (**E**) Hematoxylin & Eosin (H&E) representative images of each treatment group (left panel). Measured tumor volumes for each animal (right panel) that received control nanomiR (*n* = 7); control nanomiR and IR (*n* = 7); miR-217-5p nanomiR (*n* = 9); miR-217-5p nanomiR and IR (*n* = 10). Red bar = mean. ANOVA with a post hoc Tukey’s test used to test for significance in (**B**,**C**,**E**). * denotes *p*-value < 0.05. Original Western blot figures can be found in Supplementary File S1.

Our findings thus far predict that the in vivo delivery of miR-217-5p will hinder tumor growth and potentially cooperate with cytotoxic therapeutics. To evaluate this using a clinically translatable miRNA delivery platform, bioreducible nanoparticles [21] containing either a scrambled miRNA sequence as a control or miR-217-5p mimics were used to transfect GBM neurospheres. Nanoparticle-based delivery of miR-217-5p mimics decreased the levels of endogenous EZH2 by ~70% and H3K27me3 by ~25% (Figure 5A), significantly decreased neurosphere-forming capacity (Figure 5B), and rescued the expression of five of the six putative stemness-repressing genes (Figure 5C) shown earlier to be repressed by Oct4/Sos 2 and the PRC2 function (Figure 2D,E). To test the therapeutic potential of miR-217-5p nanomiRs, mice bearing pre-established orthotopic neurosphere-derived GBM xenografts were treated with a control or miR-217-5p nanomiRs by direct intra-tumoral infusion via a trans-cranial cannula twice per week for 2 weeks starting 6 weeks post-tumor cell implantation (eight doses in total) (Figure 5D). Two weeks after beginning nanoparticle infusions, a subset of animals began IR therapy in combination with either a control or miR-217-5p nanomiRs (Figure 5D). Tumor burden, quantified in brains collected 28 days after treatment initiation, demonstrated a significant decrease in tumor size in response to either IR treatment or the active miRNA mimic with the most profound effects occurring in the animals treated with a combination of miR-217-5p nanomiR and IR (Figure 5E). These results show that combining in vivo delivery of miR-217-5p with IR generates a cooperative anti-tumor response.

## 4. Discussion

Epigenetic mechanisms play crucial roles in cancer initiation and progression, intra-tumoral cancer cell heterogeneity, and therapeutic sensitivity. Clinically impactful examples in brain cancer include the prognostic and therapeutic roles of dysregulated DNA methylation due to oncogenic IDH mutations in low–intermediate grade gliomas and dysregulated histone modification and chromatin structure due to H3K27 mutations in diffuse midline gliomas. These and other epigenetic regulators of gene expression (both coding and non-coding RNA) function in coordination with stemness-driving and stemness-inhibiting signals to control the formation and maintenance of tumor-propagating GSCs and oncogenesis in complex ways [7,13,15]. We now show that stemness-driving signals coordinated by the oncogenic factors Oct4 and Sox2 decrease the expression of a subset of PRC2 target genes that are also repressed in clinical GBM. This inhibition in gene expression is associated with an increase in EZH2 and H3K27me3 in GSCs with high levels of EZH2 predicting decreased overall survival in GBM patients. Understanding this intricate molecular crosstalk and developing innovative approaches to block this GSC reprogramming oncogenic mechanism should yield positive therapeutic outcomes.

Identifying ways to activate the expression of the genes that suppress tumor-propagating CSCs as a strategy to treat cancer remains challenging [32]. Herein, we identified a subset of putative GSC suppressor genes that are repressed via PRC2-dependent mechanisms in GBM. We show that CAMK2B, EGR3, NPAS2, PCDH8, RGS6, and TESC are repressed by the GSC-inducing transcription factors Oct4 and Sox2. Transgenic Oct4 and Sox2 expression also increased the repressive H3K27me3 mark at the promoters of all six putative GSC-suppressive genes, and pharmacological EZH2 inhibition restored expression in all six cases. Tumor-suppressive roles have been reported for all six of these genes. CAMK2B silencing correlates with breast cancer progression and functions as a tumor suppressor in renal papillary cell carcinoma [27,33]. Loss or inactivation of either EGR3 or RGS6 correlates with a more aggressive tumor cell phenotype in multiple cancers [29,34], including GBM [35]. Circadian clock gene NPAS2 was initially described as a tumor suppressor [36] gene but emerging evidence suggests a dual nature depending on the tumor type [37], while a role in GBM has not been described previously. Similarly, PCDH8 can also drive or inhibit tumor cell phenotypes in a context-dependent manner [38,39]. Interestingly, TESC has been reported to play oncogenic roles in several cancers, but information about its function in brain tumors is unclear [40,41]. Our current findings add significantly to the growing amount of knowledge regarding the EZH2:H3K27me3 axis in the contexts of malignancy and GSC phenotype regulation, and it is predicted that blocking this axis can negatively impact GBM growth.

Drugs that function by modifying epigenetic drivers of cancer have multiple potential advantages, including (i) potential efficacy across diverse oncogenic mutational backgrounds, (ii) unique and potentially favorable toxicity profiles, and (iii) the potential to cooperate with other targeted and cytotoxic therapies. These ideas have led to considerable efforts focused on developing efficient and selective epigenetic inhibitors, including one EZH2 inhibitor, tazemetostat (EPZ-6438), which is FDA-approved for follicular lymphoma and epithelioid sarcoma [42]. Despite the promising clinical effects in peripheral tumors and encouraging observations in pre-clinical studies, tazemetostat is subject to poor brain bioavailability, limiting its impact on brain tumors [43]. New and emerging developments in nucleic acid delivery technologies, combined with approaches that focus on local drug delivery to the brain, such as convection-enhanced delivery (CED), are opening the door to the harnessing of cell biological functions inherent to specific coding and noncoding RNAs for therapeutic application aimed at addressing these shortcoming [44]. We envision our nanomiR platform to be highly compatible with emerging CED-based approaches that enable local delivery of nucleic acids, including miRNAs or siRNAs, to brain tumors to bypass the blood–brain barrier and facilitate the development of new targeted therapeutic approaches.

## 5. Conclusions

In conclusion, our research has shown that stem cell-driving events induce epigenetic events that repress subsets of miRNAs that regulate GBM cell phenotype transitions to a tumor-propagating GSC state [7,15]. We now show that one of these miRNAs, miR-217-5p, inversely associates with and regulates the GSC phenotype. We also show that miR-217-5p directly targets EZH2 and regulates the expression of the tumor-suppressive PRC2-dependent gene targets with tumor/GSC-suppressive functions (discussed above) that are also downregulated in clinical GBM. Taking advantage of our recently developed platform for in vivo miRNA delivery [13,21], we delivered miR-217-5p mimics to pre-established GBM xenografts and showed that miR-217-5p nanomiRs significantly reduced tumor growth in orthotopic GBM models and enhanced the effects of radiation therapy. These findings add to our previous reports identifying miR148a, miR-10b-5p, and miR-296-5p as therapeutically translatable miRNAs that regulate GSCs through epigenic gene targets (DNMT, TET2, HMGA1, respectively) [7,13,15,21,39]. Impactful future directions of this research include determining the optimal combinations of miRNA mimics and antimiRs to most effectively “normalize” GSC-inducing miRNA networks and epigenetic dysfunction in GBM. Our findings also highlight the potential for cooperation between epigenetic-targeting miRNAs and standard-of-care cytotoxic therapy.

## Data Availability

The data supporting this study are available within the paper and its Supplementary Data File. All other data are available from the authors upon reasonable request.

## Supplementary Materials

The following supporting information can be downloaded at: https://www.mdpi.com/article/10.3390/cancers17010080/s1, Figure S1: Gene-set enrichment analysis (GSEA) of genes differentially regulated by Oct4 and Sox2 in GBM neurospheres, Figure S2: Oct4/Sox2 represses a subset of genes associated with tumor suppression in a PRC2 dependent manner, Figure S3: miR-217-5p regulates EZH2 expression and the stem cell phenotype in GSCs, Table S1: Primers, Table S2: Antibodies; File S1: Original Western blot figures.
