# Supplementary material for: miR-217-5p NanomiRs Inhibit Glioblastoma Growth and Enhance Effects of Ionizing Radiation via EZH2 Inhibition and Epigenetic Reprogramming
Source: Cancers (Basel). 2024 Dec 30;17(1):80. doi: 10.3390/cancers17010080 (PMC11719642; doi:10.3390/cancers17010080)
Supplement: Supplementary file 1 [file cancers-17-00080-s001.zip › cancers-3361244-supplementary.pdf]

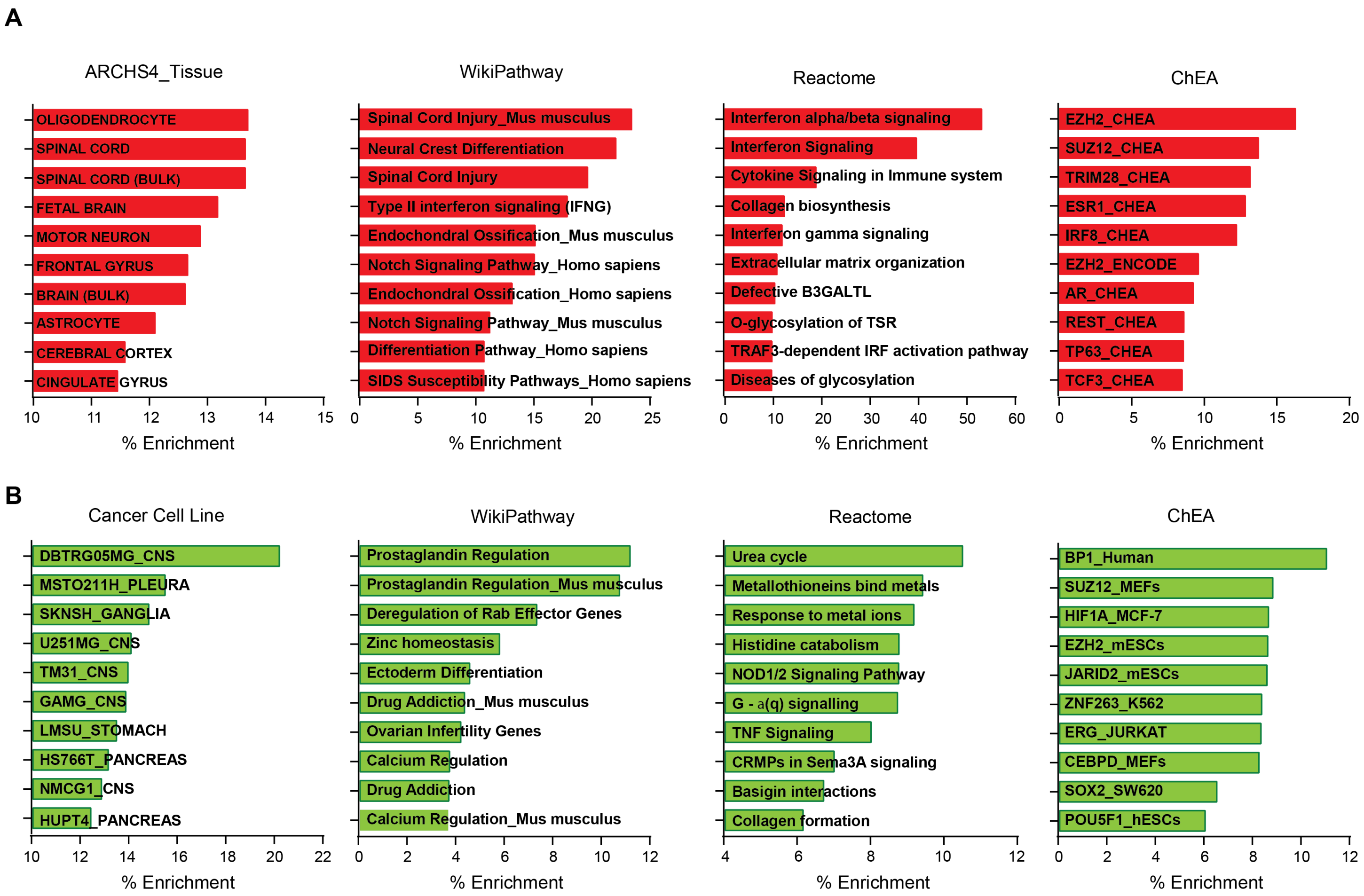

Figure S1: Gene-set enrichment analysis (GSEA) of genes differentially regulated by Oct4 and Sox2 in GBM neurospheres. RNA-Sequencing was performed in 2 distinct GBM neurosphere isolates with or without transgenic expression of Oct4 and Sox2. This analysis uncovered 1404 up-regulated and 1435 down-regulated genes that changed significantly in 2 distinct GBM neurosphere models (p-adj <0.05, log fold change >1 or < -1). Nest we performed GSEA using the Enrichr online platform (https://maayanlab.cloud/Enrichr/) to identify pathways depleted **(A)** or enriched **(B)** under these conditions.

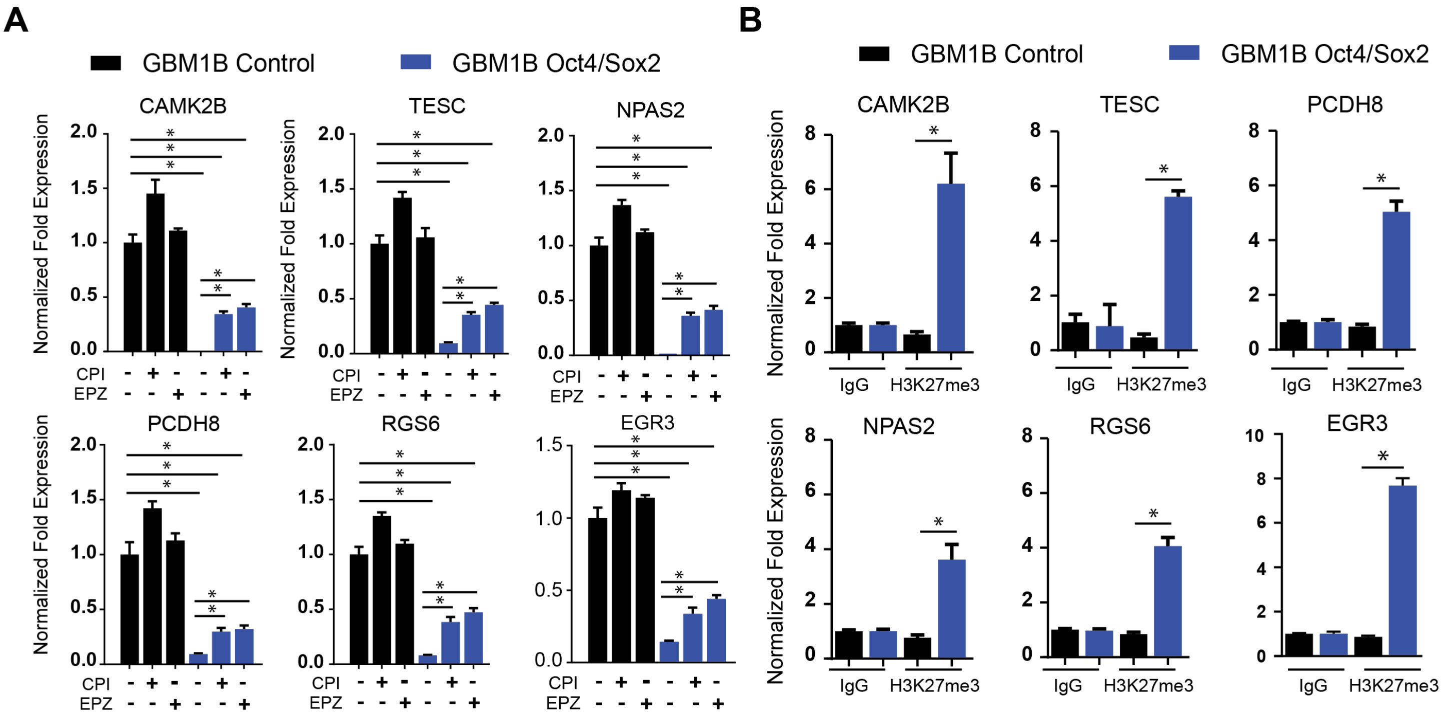

Figure S2. Oct4/Sox2 represses a subset of genes associated with tumor suppression in a PRC2 dependent manner. (A) qRT-PCR for predicted PRC2 targets with and without the EZH2 inhibitors CPI and EPZ in control vs Oct4/Sox2 over expression in the primary GBM cell line GBM1B. **(B)** ChIP-PCR for H3K27me3 at the promotor region for six punitive PRC2 targets in GSCs expressing transgenic Oct4 and Sox2. GSCs expressing GFP were used as controls. ANOVA with a post-hoc Tukey's test used to test for significance in panel A and two sample t-test used to test for differences in B. * denotes p-value<0.05.

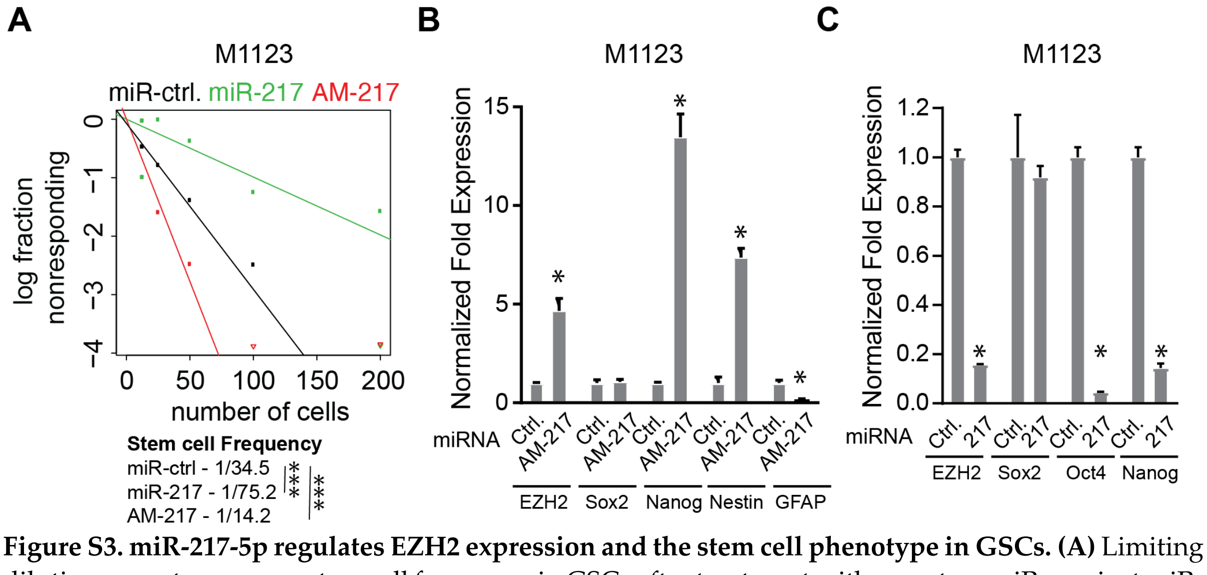

Figure S3. miR-217-5p regulates EZH2 expression and the stem cell phenotype in GSCs. (A) Limiting dilution assay to measure stem cell frequency in GSCs after treatment with an antagomiRs against miR-217-5p (AM-217) or miR-217-5p mimics (217). qRT-PCR expression of EZH2, stem cell and neuronal lineage markers 5 days after treatment with miR-217-5p antagomiR **(B)** or a miR-217-5p mimic **(C).** Two sample t-test used to test for differences in B and C. * denotes p-value<0.05.

Table S1: qRT-PCR primers.

Stem cell and Lineage markers

Target	Forward	Reverse
18S	ACA GGA TTG ACA GAT TGA TAG CTC	CAA ATC GCT CCA CCA ACT AAG AA
NESTIN	AAG ACT TCC CTC AGC TTT CAG	AGC AAA GAT CCA AGA CGC C
BMI1	AAT CCC CAC CTG ATG TGT GT	GCT GGT CTC CAG GTA ACG AA
O4	CTA CTG CTC TGG GTC CCA GG	CTG CCA CTG AAC CGA GAT GG
TUJ1	CAA CAG CAC GGC CAT CCA GG	CTT GGG GCC CTG GGC CTC CGA
GFAP	GGC AAA AGC ACC AAA GAC GG	GGC GGC GTT CCA TTT ACA AT
SOX2	GCC GAG TGG AAA CTT TTG TCG	GGC AGC GTG TAC TTA TCC TTC T
Oct4	TGA ACT GTG GTG GAG AGT GC	AGG AAG GGC TAG GAC CAG AG
NANOG	CTA AGA GGT GGC AGA AAA ACA	CTG GTG GTA GGA AGA GTA AAG G
CD133	AGT CGG AAA CT GCA GAT AGC	GGT AGT GTT GTA CTG GGC CAA T
CD44	CTG CCG CTT TGC AGG TG TA	CAT TGT GGG CAA GGT GCT ATT
KLF4	CCC ACA TGA AGC GAC TTC CC	CAG GTC CAG GAG ATC GTT GAA
OLIG2	AAT ACC GTT ATG GAC TCG GAC GCC	ATT GTC GAC TCA CTT GGC GTC GGA

Chromatin Immunoprecipitation (ChIP)

Target	Forward	Reverse
CAMK2B	GAG GAT GAA GGC GTC AGT ACA	GAC ACG CTG CTC CTG CAC
EGR3	GAG GAG CTT CCA TTG TGA CG	TCC TAG CAA GCT CAC TGC TG
NPAS2	GTC CAC TTG CCT CTC CTC TG	CCA GCC TGG GAC AAG CAC
PCDH8	CTC TAC ATG CAGGGT GCTGA	TTT ATA GCC GCC GAT ATG CT
RGS6	TTC TGC TTC CCC CAA GAA TA	ACC GTC AGC GAT ATT GTT CC
TESC	GCT CGC TGA CTT CCT TGA CT	CTC ACG TAC CTC GTC CCT TC

PRC2 Targets

Target	Forward	Reverse
CAMK2B	CTC TAC GAG GAT ATT GGC AAG GG	GCT TCT GGT GAT CTC TGG CTG
DGKI	GCA GGT CTC GTA CAG GAA AGC	CAC TCC AGT CCA AAG TCG CTC
EGR3	GAC ATC GGT CTC ACC AAC GAG	GGC GAA CTT TCC CAA GTA GGT
LHX2	ATG CTG TTC CAC AGT CTG TCG	GCA TGG TGC TCT CGC TGT
MN1	CCT CCC GGA CTT CCA CAGT	GGC TCC TTG GTT CGT CAC C
NELL1	TAT GAG CGT GTG ATA GAC CCTC	TCC CAT CTT GGA TGA TCC CTT
NPAS2	ACA CCC TTT CAA GAC CTT GCC	AGG TTC GTC AAC TAT GCA CAT TT
PAQR9	CAA GAG CCG TAC CGA CTG G	CCG ATA ATG TCG AAA AGA CCC G
PLDH17	GCA CGG TGA TCG GGA ACAT	GCG CTG CTT GGT GTA GAG G
PCDH8	TAT GGG CAC GAG CAC TTCC	CCA GCG TCA AGT TGT ACT CGG
RGS6	ACT TAG CAA GAC TCC AGA GGG	GAC ATC CCA AAA GGC TCG TTC
SOX 1	CAG TAC AGC CCC ATC TCC AAC	GCG GGC AAG TAC ATG CTGA
SOX 7	AGC CGG AGC AGA CCT TCTT	GCC GGG GAG TAA TAG GCAG
TESC	GTC GGG AAA CCC TCA CAT CG	CGA AGG TGA TCC CCT CGT ACA
WNT7A	CTG TGG CTG CGA CAA AGA GAA	GCC GTG GCA CTT ACA TTCC

PCR primers used to amplify pre-miRNAs

Target	Forward	Reverse
mir-124	TCCGTGTTCACAAGCGGAC	CATTCACCGCGTGCCTTA
mir-217	GATACTGCATCAGGAACTGATTG	GGCAATGCATTAGGAACTGAT
U6	CTCGCTTCGGCAGCACA	AACGCTTCACGAATTTGCGT

Table S2: Anibodies.

Target	Company	Catalog #	Dilution
EZH2	Cell Signling	5246	1:1,000
Tri-Methyl-Histone H3 (Lys27) (C36B11)	Cell Signling	9733	1:1,000
Tri-Methyl-Histone H3 (Lys4) (C42D8)	Cell Signling	9751	1:1,000
Tri-Methyl-Histone H3 (Lys9) (D4W1U)	Cell Signling	13969	1:1,000
Tri-Methyl-Histone H3 (Lys36) (D5A7)	Cell Signling	4909	1:1,000
Histone H3 (D1H2)	Cell Signling	4499	1:1,000
Histone H3K27me3	Active Motif	39155	2 µg per ChIP
Oct4	Cell Signaling	2750	1:1,000
Sox2	Cell Signaling	3579	1:1,000
GAPDH	SANTA CRUZ	SC-47724	1:1,000
bACTIN	Millipore Sigma	A2066-100UL	1:1,000
