# Supplementary figures and images for: miR-217-5p NanomiRs Inhibit Glioblastoma Growth and Enhance Effects of Ionizing Radiation via EZH2 Inhibition and Epigenetic Reprogramming
Source: Cancers (Basel). 2024 Dec 30;17(1):80. doi: 10.3390/cancers17010080 (PMC11719642; doi:10.3390/cancers17010080)

Figure 1:

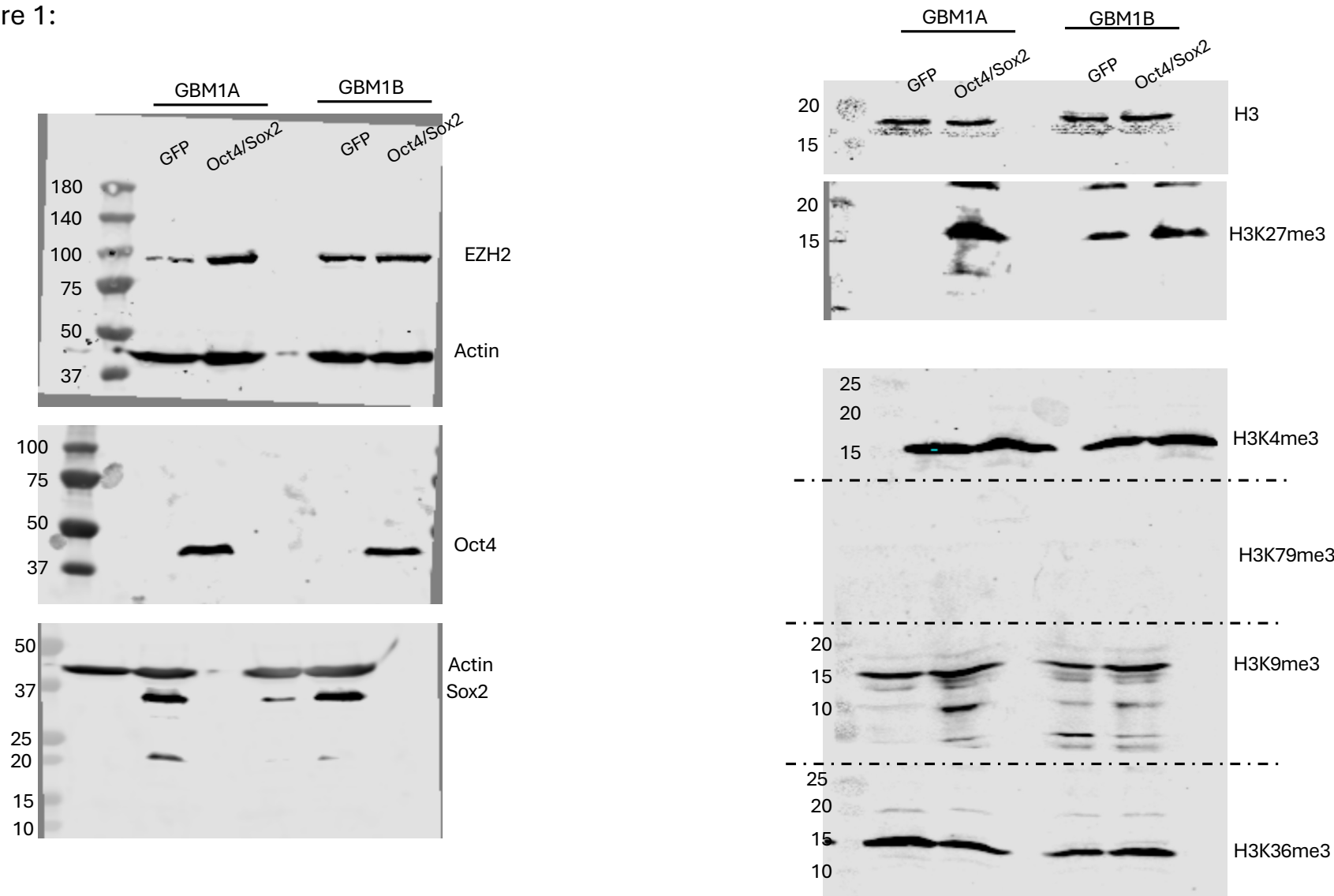

Figure 3:

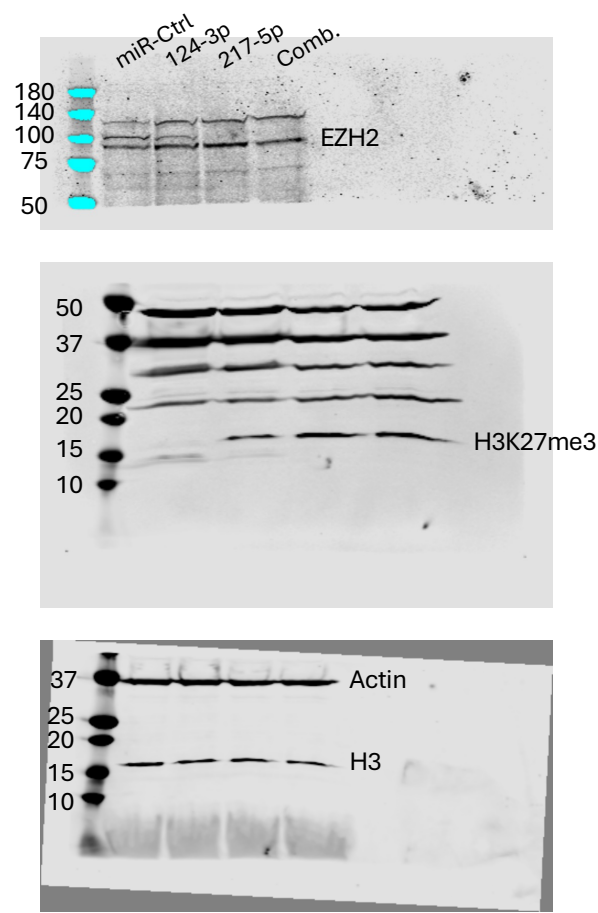

Figure 4:

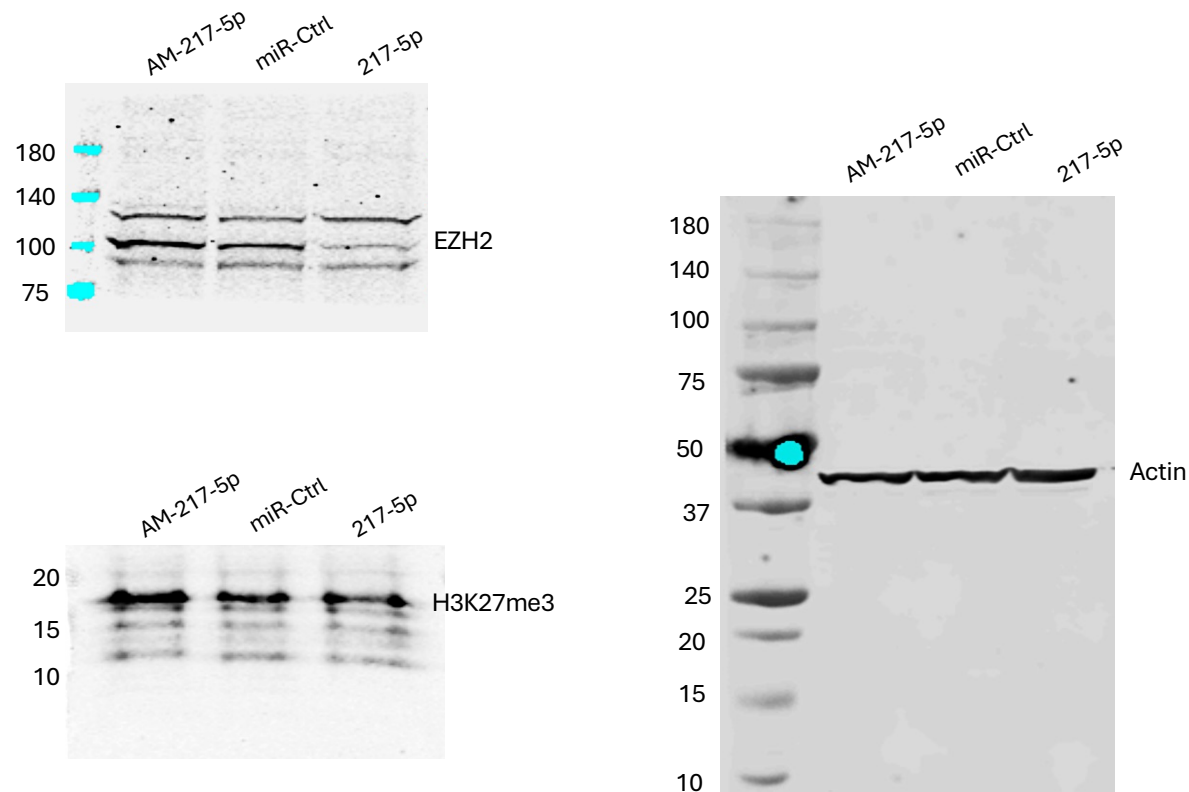

Figure 5:

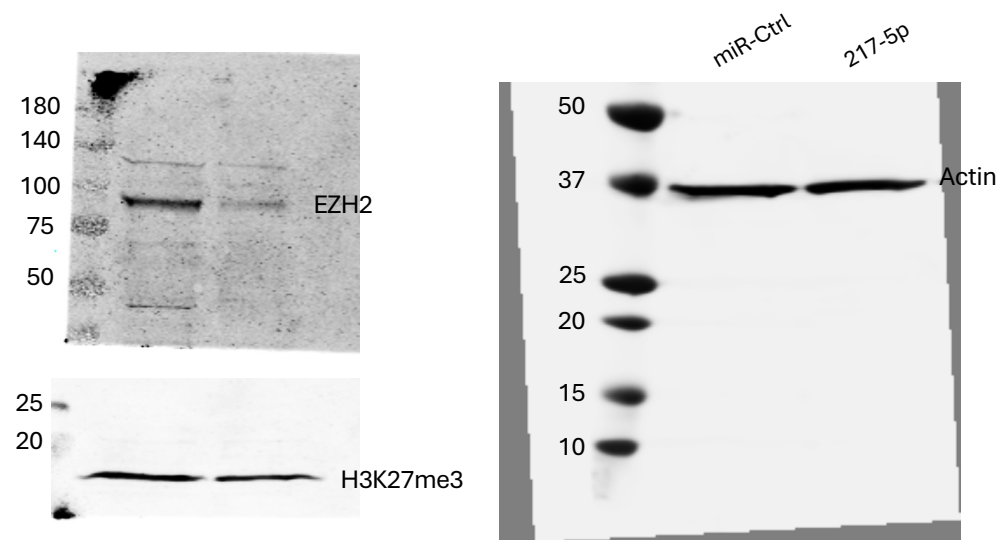

Supplement: Supplementary file 1 [file cancers-17-00080-s001.zip › cancers-3361244-File S1. Original Western blot figures.pdf]
